# NMR-based metabolomics to select a surgical method for treating papillary thyroid carcinoma

**DOI:** 10.6061/clinics/2018/e333

**Published:** 2018-11-06

**Authors:** Bo Wang, Li-Yong Zhang, Si-Si Wang, Ying-Hong Yang, Wen-Xin Zhao

**Affiliations:** Fujian Medical University Union Hospital, Fuzhou, PR, China

**Keywords:** Thyroid Papillary Carcinoma, Metabolomics, Principal Component Analysis, Total Thyroidectomy, Unilateral Thyroidectomy

## Abstract

**OBJECTIVE:**

This study aims to investigate differences in the metabolomic profiles of patients who received different surgeries for papillary thyroid carcinoma (PTC).

**METHODS:**

Two surgical methods, i.e., unilateral and total thyroidectomy, were employed according to different disease conditions. Sera from patients who were treated with levothyroxine sodium tablets before and after surgery was analyzed with a Bruker 500 Hz nuclear magnetic resonance (NMR) spectrometer. Data were analyzed via principal component analysis (PCA) and partial least squares discriminate analysis (PLS-DA) with SIMCA-P+ 11.0 software, and metabolites were obtained and compared. The first and second principal components were selected from PCA, PLS-DA, and orthogonal partial least squares discriminate analysis (OPLS-DA). A *p-*value less than 0.05 was considered statistically significant.

**RESULTS:**

There were significant differences in serum metabolomics before and after surgery. Compared with unilateral thyroidectomy, total thyroidectomy reversed some highly increased metabolite levels (e.g., taurine and betaine). More significant variations in abnormal metabolites were noted after total thyroidectomy than after unilateral thyroidectomy (e.g., alanine, choline, hippurate, and formic acid).

**CONCLUSIONS:**

The choice of surgical method for PTC patients should be based not only on the tumor condition but also on the potential consequences of metabolic variations. Total thyroidectomy reversed some increased metabolite levels but led to accumulation of some other metabolites due to the loss of thyroid function; thus, metabolic disturbances caused by thyroid hormone deficiency should be prevented in advance.

## INTRODUCTION

Thyroid carcinoma is the most common malignancy of the endocrine system, accounting for 2% of all systemic tumors. Papillary thyroid carcinoma (PTC) accounts for 65-75% of thyroid cancers 1. In addition, approximately 20% of patients demonstrate recurrence after treatment, of which approximately 8% died due to local recurrence 2. Therefore, the first surgical protocol for PTC is vital, and a standardized and thorough first treatment is directly associated with the probability of patient survival 3.

At present, surgical resection is the most efficient therapeutic method for PTC. Total thyroidectomy and unilateral thyroidectomy are currently two of the main kinds of thyroidectomy procedures. Compared with unilateral thyroidectomy, total thyroidectomy can effectively prevent residual cancer and reduce the recurrence rate of occult carcinoma. Furthermore, total thyroidectomy can be used to treat postoperative recurrence and metastasis 4. However, loss of thyroid function can lead to a variety of postoperative complications. Therefore, artificial thyroxine supplementation is generally used as an alternative treatment after total thyroidectomy. Partial retention of the unilateral thyroid gland may prevent the occurrence of postoperative loss of thyroid function 5.

In addition, a wide range of concentrations of serum thyroxine (T4), free thyroxine (FT4), and thyroid-stimulating hormone (TSH) have been reported in patients with differentiated thyroid carcinoma after surgery, and the levels of these metabolites have a direct relationship with thyroidectomy 6. Metabolomic analysis has been successfully applied to identify tumor markers in breast and prostate cancers. In addition, nuclear magnetic resonance (NMR) spectroscopy and mass spectrometry (MS) are extensively utilized for metabolomic analysis. Without isolating samples, differences in relaxation behavior on NMR and metabolite dispersion are used to isolate different signals by exerting changes in metabolic profile techniques 8. All metabolites in a sample are detected with the same sensitivity, and bioinformatic, chemometrics, and statistical analysis are then performed to evaluate the metabolite data. Additionally, it has been revealed that small-molecule metabolic marker levels are significantly increased in cancer cells. Furthermore, metabolic changes are closely related to intercellular communication, energy utilization, and cell proliferation 10.

In this study, metabolomics methods were utilized to study the differences in serum metabolites in patients with PTC treated with different surgical methods. Moreover, the effects of different surgical methods on the postoperative and therapeutic outcomes in patients with PTC were investigated.

## MATERIALS AND METHODS

### Study design

Forty patients with PTC were equally divided into two groups. In the first group (L group), twenty patients, including 8 males and 12 females with a mean age of 39.50±9.72 years old, were treated with unilateral thyroidectomy; in the second group (T group), twenty patients, including 6 males and 14 females with a mean age of 40.45±10.63 years old, were treated with total thyroidectomy. Two timepoints were set for each group, namely, L1 (before surgery) and L2 (after surgery) for the L group and T1 (before surgery) and T2 (after surgery) for the T group.

The study protocols were approved by the Institutional Medical Ethics Review Board of Fujian Medical University Union Hospital in Fuzhou, China. Informed consent was obtained from each patient. Samples were collected 24-48 h before surgery and within one week after surgery and were stored at -80°C.

The inclusion criteria were as follows: (1) at least one side of the thyroid after lobectomy was pathologically confirmed with PTC; (2) no cervical lymph nodes or distant metastasis were present; (3) after surgery, levothyroxine sodium tablets (L-T4) were regularly consumed over 3 months; (4) TSH levels were less than 0.1 IU/L; and (5) free triiodothyronine (FT3) and free thyroxine (FT4) levels were normal.

The exclusion criteria were as follows: (1) antitumor treatment for 4 weeks before surgery; (2) chronic lymphocytic thyroiditis or thyroid dysfunction; (3) PTC patients with distant metastasis; (4) hypertension, diabetes, and other metabolic diseases, or other nervous system disorders affecting metabolism; (5) abnormal liver or kidney function; (6) other malignancies; (7) long-term utilization of drugs affecting metabolism; (8) vegetarian diet; and (9) women who were pregnant or lactating.

### Detection of NMR profiles

For each sample, serum (400 μl), phosphate-buffered saline (PBS) (100 μl), and deuterium oxide (D2O) (100 μl) were mixed and then centrifuged at 12000 rpm for 10 min at 4°C. The liquid supernatant (500 μl) was collected as an NMR detector. The NMR profile was obtained by NMR spectroscopy with a D2O lock and 5-mm triple HCN resonance probe at a temperature of 300 K and spectral width of 5 kHz. In this study, a nuclear Overhauser effect spectroscopy with presaturation Carr–Purcell–Meiboom–Gill (NOEPR-CPMG) impulse sequence was used for data sampling. A presaturation module was utilized to suppress the water peak signal for 2 s, while a cyclotron wave module was used to suppress the broad peaks caused by proteins and lipoproteins. In addition, the signal was accumulated 128 times. Furthermore, D2O was utilized as an internal standard to obtain the sample spectrum.

### Data processing and statistical analysis

After phase-position and baseline one-dimensional 1H NMR spectra were collected with Bruker TopSpin software (Bruker, Switzerland), lactate was used for calibration with two peaks in the high field (1.33 ppm). The calibrated spectra were integrated in a 0.50-9.0 ppm segment using AMIX software, version 39.11 (Bruker, Switzerland), and the integration range was 0.005 ppm. To eliminate the effect of the residual water peak, for each peak, the 4.315-6.65 ppm segment containing the residual water peak and urea peak signals was removed. The NMR spectra were processed with the area-normalization method. The variation in the area of each metabolite was normalized using an average peak derived from normal serum. The normalized data were imported into the SIMCA-P+ 11.0 software package (Umetrics, Basel, Switzerland) for multivariate analysis. The collected data were evaluated with principal component analysis (PCA) and partial least squares-discriminate analysis (PLS-DA). Through orthogonal partial least squares discriminant analysis (OPLS-DA), metabolites with statistical significance were further summarized by determining a correlation coefficient for each metabolite. Additionally, after comparing the corresponding correlation coefficient with a table representing threshold values, metabolites that showed differences between the groups were also evaluated. The first and second principal components were selected from PCA, PLS-DA, and OPLS-DA. In this study, *p<*0.05 was considered statistically significant.

## RESULTS

### Total thyroidectomy led to different metabolite outcomes compared with unilateral thyroidectomy

**Figure 1:**
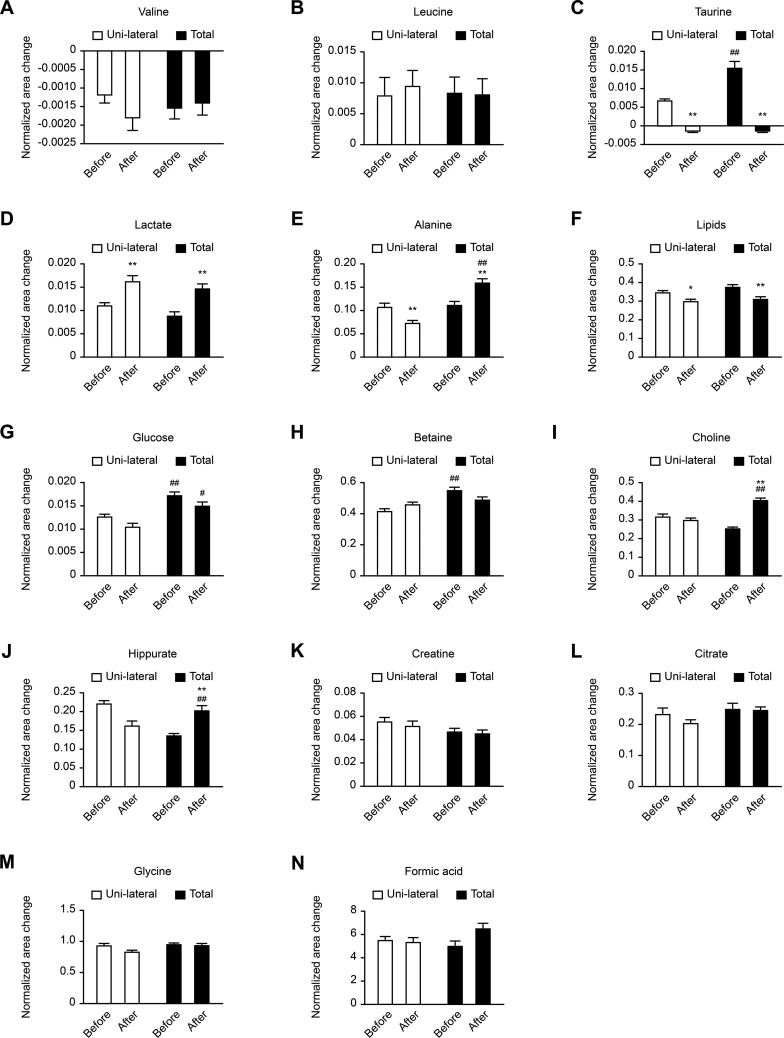
Comparison of metabolites among different groups (or subgroups). The left of the X-axis denotes the unilateral thyroidectomy (L) groups, and the right one denotes the total thyroidectomy (T) groups. In addition, *p<0.05 and **p<0.01 represent a comparison of surgery *vs*. before surgery, and ^#^*p*<0.05 and ^##^*p*<0.01 represent a comparison of the T *vs*. L groups.

One-dimensional 1H NMR spectra of samples were calibrated (1.33 ppm) with lactate in the high-field after phase and baseline correction with Topspin software. Comparisons of the magnetic numbers among different groups were performed, and the results are shown in Figure 1; the metabolites were identified in a previous study 11.

Compared with patients treated with unilateral thyroidectomy, those treated with total thyroidectomy had distinct metabolomic variations (Figure 1). The results are as follows. The plasma levels of valine, leucine, creatine, citrate, glycine, and formic acid were unaffected in both groups before and after surgery, and no significant differences were observed between the two groups of patients who underwent different surgeries. The concentrations of taurine, glucose, and betaine were remarkably higher in patients who underwent total thyroidectomy than in those who underwent unilateral thyroidectomy. Both types of surgery significantly decreased taurine levels to below the normal level. On the one hand, the same attenuating influences of both types of thyroidectomy were found on lipid and glucose levels; however, glucose levels were always significantly higher after total thyroidectomy than after unilateral thyroidectomy. On the other hand, both types of surgery led to increased lactate levels. However, more distinct trends were observed for other metabolites. For instance, alanine levels declined in the L group after surgery but increased in the T group. Similar variations were found in choline, hippuric acid, and formic acid as. These findings demonstrate that total thyroidectomy caused different significant metabolomic effects compared with unilateral thyroidectomy, which may particularly have aggravated some metabolic disturbances in patients.

**Figure 2:**
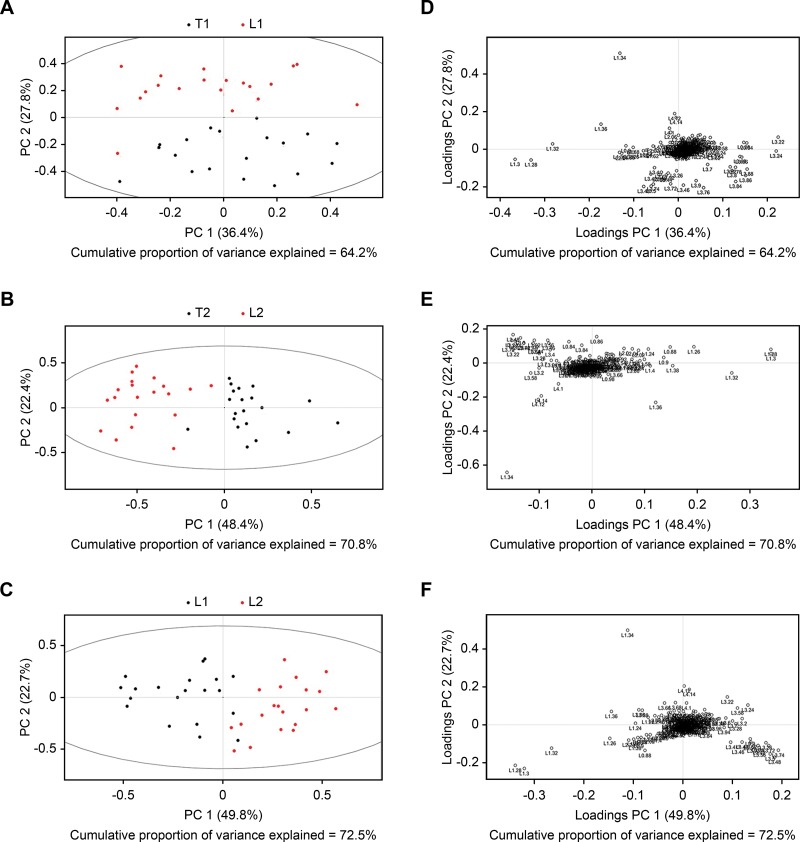
Unit variance scaling of PCA plots. A PCA score plot (A) and PCA loading plot (B) were obtained from the T1 and L1 groups (A), T2 and L2 groups (B), and L1 and L2 groups (C) based on 1H NOESYGPPR1d NMR spectra of serum. Corresponding PCA loading plots were obtained from the T1 and L1 groups (D), T2 and L2 groups (E), and L1 and L2 groups (F).

**Figure 3:**
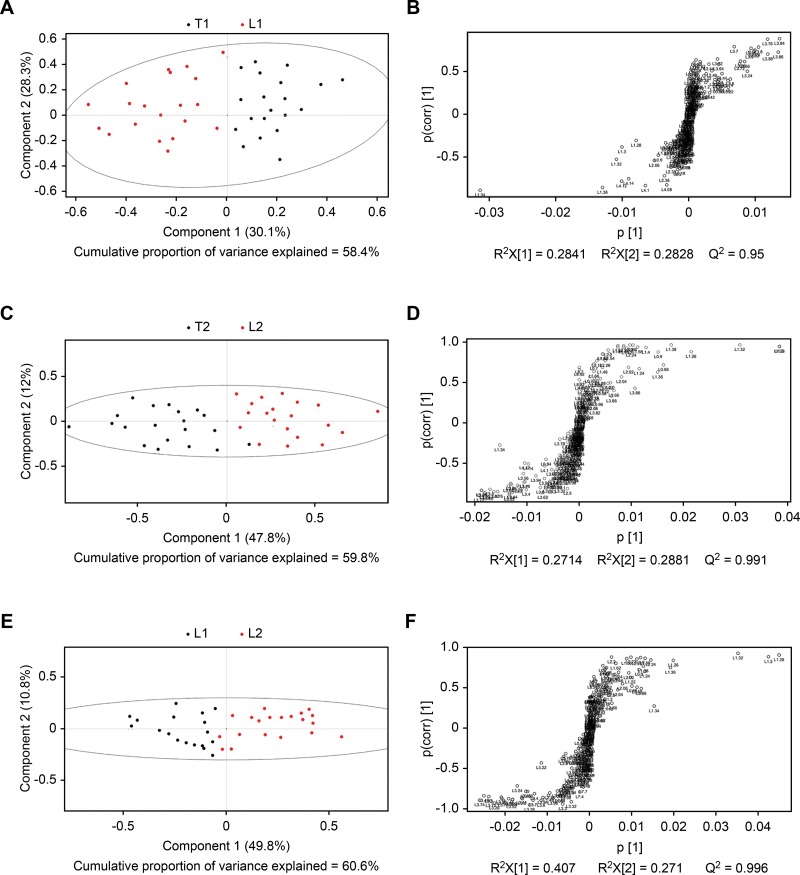
Unit variance scaling of PLS plots. A PLS score plot (A) and PLS loading plot (B) were obtained from the T1 and L1 groups (A), T2 and L2 groups (B), and L1 and L2 groups (C) based on 1H NOESYGPPR1d NMR spectra of serum. Corresponding PLS loading plots were obtained from the T1 and L1 groups (D), T2 and L2 groups (E), and L1 and L2 groups (F).

**Figure 4:**
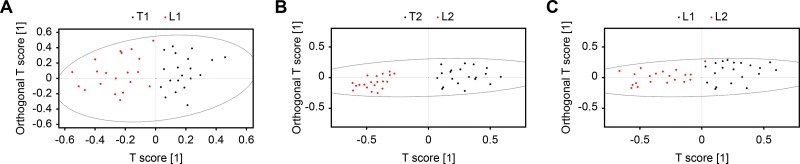
Unit variance scaling of OPLS-DA plots. OPLS-DA score plot of the T1 and L1 groups (A), T2 and L2 groups (B), and L1 and L2 groups (C).

SIMCA-P+ 11.0 software was used for multivariate pattern recognition analysis of normalized data. Data conversion was performed with the aid of unit variance (UV) scaling, and converted data were used for PCA (Figure 2), PLS-DA (Figure 3), and OPLS-DA (Figure 4). As shown in Figures 2A and 2D, there was a significant difference in the distribution of PCA scores between the T1 and L1 groups. In addition, the scores of the T1 group were mainly concentrated in the third and fourth quadrants, while those of the L1 group were focused in the first and second quadrants. In addition, the T1 group showed proper aggregation, while L1 group had clear outliers. Similarly, as illustrated in Figures 2B and 2E, there was a significant difference in the distribution of PCA scores between the T2 and L2 groups. The scores of the T2 group were mainly focused in the first and fourth quadrants, while those of the L2 group were mainly distributed in the second and third quadrants. Both groups demonstrated appropriate aggregation. As depicted in Figures 3C and 3F, a significant difference was found in the distribution of PCA scores between the L1 and L2 groups. The scores of the L2 group was mainly distributed in the first and fourth quadrants, while those of the L1 group were located in the second and third quadrants. Both groups showed appropriate aggregation; however, the L1 group presented more outliers. In comparison with the results of the PLS-DA and OPLS-DA analyses, there were significant differences between the T1 and L1, T2 and L2, and L1 and L2 groups.

## DISCUSSION

Currently, surgical resection is one of the main methods for effective treatment of PTC. Selecting the first surgery for patients with PTC has a significant influence on recurrence, residual tumor burden, and postoperative recovery. The appropriate surgical method (total thyroidectomy and unilateral thyroidectomy) for PTC is currently controversial. The thyroid is a vital endocrine organ that secretes thyroid hormones, which play crucial roles in tissue and organ metabolism 12. Thyroid surgery can trigger endocrine and metabolic disorders and induce organism-related metabolic disorders. In this study, an NMR technique was employed to detect and analyze serum levels of metabolites before and after surgery with different surgical methods. Metabolomic differences under TSH-suppression conditions were used to evaluate the effects of different surgical methods and standardization of PTC operative treatments.

Several metabolites were affected by different surgical treatment methods. For instance, lactic acid is produced through anaerobic glycolysis. Approximately 20% of the available glucose in healthy individuals is made in the lactic acid cycle, while this value is approximately 50% in patients with metabolic disorders. However, glucose generation from alanine and glycerol has been shown to be increased in cancer patients 18. Alternative thyroid hormone therapy was performed after thyroidectomy. Long-term subclinical hyperthyroidism and a relatively high metabolism in patients causes mismatching of oxygen supply and oxygen demand and leads to tissue hypoxia. The glycolysis product pyruvic acid cannot be used in the tricarboxylic acid cycle and is metabolized to lactic acid; thus, lactic acid levels were increased after surgery. In the L1 group, patients may have had small nodules or occult cancer in the contralateral residual thyroid lobe, and anaerobic glycolysis may have been caused by tumor factors. Aerobic metabolism was relatively increased in this postsurgical group, and there was greater inhibition of anaerobic glycolysis in tumors after surgery than before surgery. Another metabolite, taurine, was also significantly affected by surgery. Abnormal protein metabolism is attributed to synthetization and decomposition of protein, abnormal amino acid spectra of plasma, and a negative nitrogen balance *in vivo*. Amino acids reflect protein metabolism disorders in cancer patients 19. Taurine levels in the L2 group were lower than those in the L1 group. Taurine is not a component of protein. However, it is a conditionally essential amino acid with a wide range of physiological functions. Taurine levels largely depend on liver metabolism, and taurine combines with bile acid to form taurocholic acid. The glutathione system is an important detoxification system in the liver. The lower level of taurine in the L2 group probably represents less liver and kidney damage in these patients than in those in the L2 group, which is induced by high taurine levels to some extent.

Abnormal glucose metabolism was terminated more completely in the L2 group than in the L1 group, and termination of this metabolic pathway inhibited tumor proliferation. In addition, TSH suppression therapy had an impact on metabolism, and abnormal metabolites were increased (e.g., taurine and glucose). Thus, the proposed therapeutic method used in the L2 group better controlled postoperative TSH suppression therapy and decreased the occurrence of side effects associated with TSH suppression therapy than the treatments used in the other groups.

Several metabolite abnormalities were attenuated in the L groups and aggravated in the T groups. This suggests that patients with metabolic disorders may have worsening symptoms after total thyroidectomy. Overall, selecting one of the two mentioned surgical methods for patients with PTC should not only be based on the tumor conditions but also on the potential consequences of metabolic variations. Total thyroidectomy reversed some highly increased metabolites but simultaneously increased the accumulation of other metabolites due to the loss of thyroid function compared with unilateral thyroidectomy. Total thyroidectomy prevented tumor recurrence; however, the metabolic disturbance caused by thyroid hormone deficiency should be prevented in advance of this treatment.

## AUTHOR CONTRIBUTIONS

Wang B wrote and revised the manuscript. Zhao WX designed the study. Zhang LY, Wang SS and Yang YH were responsible for the patient care, technical advice, data acquisition, statistical analysis, interpretation of the data and composition of the manuscript. All the authors read and approved the final version of the manuscript.
